# Risk factors of birth asphyxia among newborns in public hospitals of Central Zone, Tigray, Ethiopia 2018

**DOI:** 10.1186/s13104-018-3611-3

**Published:** 2018-07-20

**Authors:** Hagos Tasew, Micheal Zemicheal, Girmay Teklay, Teklewoini Mariye, Ebud Ayele

**Affiliations:** 1 School of Nursing, College of Health Science and Comprehensive Specialized Hospital, Aksum University, Tigray, Ethiopia; 2 School of Medicine, College of Health Science and Comprehensive Specialized Hospital, Aksum University, Tigray, Ethiopia

**Keywords:** Birth asphyxia, Risk factors, Neonates, Central Zone, Tigray, Ethiopia

## Abstract

**Objective:**

The aim of study was to identify risk factors of birth asphyxia among newborns in public hospitals of Central Zone Tigray, Ethiopia 2018.

**Results:**

A total of 88 cases and 176 controls were included in the study. Thirty (34.1%) cases and 28 (15.9%) of controls were not able to read and write. Twenty-one (23.9%) cases and 9 (5.1%) controls were had meconium stained on pelvic examination. Multivariable logistic regression analysis showed that maternal illiteracy [AOR = 6; 95% CI (1.51, 23.80)], low birth weight [AOR = 6.9; 95% CI (3.01, 15.81)], preterm [AOR = 2.2; 95% CI (1.022, 4.76)], prim parous [AOR = 3.1; 95% CI (1.51, 6.38)], antepartum hemorrhage [AOR = 12; 95% CI (2.29, 63.11)] and meconium stained amniotic fluid [AOR = 7.88; 95% CI (2.92, 21.29)] were independent risk factors of birth asphyxia.

## Introduction

Birth asphyxia is defined as a failure to initiate, establish and sustain breathing at birth. It can also be defined as placental or pulmonary gas exchange impairment leading to hypoxemia and hypercarbia [1, 2]. Birth asphyxia is oxygen deficit at delivery which can lead to severe hypoxic organ damage (heart, lungs, liver, gut, kidneys), but brain damage is of most concern and perhaps the least likely to quickly or completely heal. In more pronounced cases, an infant will survive, but with damage to the brain manifested as either mental, such as developmental delay or intellectual disability, or physical, such as spasticity [3, 4].

A diagnosis of birth asphyxia may be made when a baby has a < 7 Apgar score. Another way of identifying birth asphyxia is checking the acidity of the blood in the umbilical cord. If it is too acidic, it can be a sign that the baby has had a period of oxygen deprivation [5]. A baby diagnosed with birth asphyxia may be breathing weakly or not breathing at all; it may have bluish or very pale skin, a low heart rate, poor muscle tone or be experiencing seizures a few hours after birth [1, 6].

Over 130 million infants born every year globally and about four million neonatal deaths occurred each year [7]. Neonatal deaths (deaths in the first 28 days of life) account for almost 40% of under-five deaths and 29/1000 neonatal death occur in Ethiopia [8, 9]. For over three quarters of these deaths was due to serious infections, including tetanus (36%), complications of preterm birth (27%) and birth asphyxia (23%) in developed countries [10–13]. About 1 million babies were died due to birth asphyxia related complications in the 1st month of life, and millions have a lifetime of impairment. Birth asphyxia was one of the contributors of early neonatal death with 34% and followed by 25% prematurity and 18% sepsis and other infectious conditions [9, 14].

There are tested risk factors of birth asphyxia in different studies however there are factors which are not tested particularly in our setting [15]. Hence, to reduce the impact of birth asphyxia on neonatal morbidity and mortality it needs further different study in diverse setting. Therefore, this study aims to identify the risk factors of birth asphyxia which produce broader implication on prevention of birth asphyxia.

## Main text

### Study area and period

The study was carried out in public hospitals of Central Zone Tigray, Ethiopia. Data collection for this study was undertaken from January up to February 2018.

### Study design

A facility based unmatched retrospective case–control study design was employed.

### Source population

The source population was all mothers were born their child in central zone, Tigray, Ethiopia.

### Study population

#### For cases

Mothers with their newborns who diagnosis as birth asphyxia.

#### For controls

Mothers with their newborns who diagnosis without birth asphyxia.

### Sample size calculation

Sample size of the study was calculated using EPI Info software version 7.1.1 with the following parameters for unmatched case control study: confidence level = 95%; power = 80%; odds ratio = 2.53; case to control ratio = 1:2; proportion of controls with exposure 17.7% [16]; proportion of cases with exposure = 35.2%. Assuming a non-response rate of 10% the sample size for cases = 88; sample size for controls = 176 the overall sample size was = 264.

### Sampling technique

Systematic random sampling technique was used to select the study subjects from four public hospitals with every two study subjects for both cases and controls.

### Study variables

- Dependent variable
  - Birth asphyxia
- Independent variables
  - Antepartum factors
  - Intrapartum factors
  - Fetal factors

### Operational definitions

#### Birth asphyxia

When the newborn has at least one of the following signs, not breathing, gasping, < 30 breaths per minute or < 7 APGAR score [6].

#### Low birth weight

When the newborn weight was < 2500 g [17].

#### Preterm

When the newborn was born < 37 gestational age [18].

### Data collection tool and procedure

Data was collected using interviewer administered structured questionnaire adapted [19], observational and chart analysis. The questionnaire was initially prepared in English and then translated into Tigrigna. The questioner reliability was checked using Cronbach’s alpha.

### Data quality control

Quality of the data was assured with properly designed data collection instruments. The enumerators and the supervisors were given training for 5 days on procedures, techniques and ways of collecting the data. Five percent pretest was done at Shul hospital to check consistency of the questioner. The collected data was reviewed and checked for completeness by principal investigator and co-investigators weekly.

### Plan for data processing and analysis

Data was entered and cleaned using Epi info version 7.1.1. Data was analyzed using SPSS version 22.0 statistical software. Cross tabulation was done among dependent variable and independent variables. Binary logistic regression model was used to determine significant association between birth asphyxia and possible risk factors. Variables which were show statistical significance during bivariate analysis at p-value ≤ 0.25 were entered to multivariable logistic regression. Multivariable logistic regression was done with considering a selected variables of bivariate logistic regression analysis. Data was finally presented and interpreted at p-value < 0.05 being considered as statistically significant.

### Results

#### Socio-demographic characteristics of study participants

In this study, a total of 88 neonates who had birth asphyxia (cases) with their index mothers and 176 newborns who had no birth asphyxia (controls) with their index mothers were included making a response rate of 100%. Forty-nine (55.7%) of cases and 64 (36.4%) controls were living in rural areas. Regarding to marital status, 75 (85.2%) cases and 166 (94.3%) of controls were married. Forty-seven (53.4%) of cases and 89 (50.6%) of controls were house wives and 30 (34.1%) cases and 28 (15.9%) of controls were not able to read and write (Table 1).

**Table 1:** Distribution of socio demographic characteristics of cases and controls attending public hospitals of Central zone, Tigray Region, North Ethiopia 2018

Variables	Category	Cases	Controls	Total
		n = 88 (%)	n = 176 (%)	n = 264 (%)
Religion	Orthodox	76 (85.4)	150 (85.4)	226 (85.6)
	Muslim and others	12 (13.63)	26 (14.7)	38 (14.4)
Residence	Urban	64 (36.4)	112 (63.6)	151 (57.2)
	Rural	49 (55.7)	64 (36.4)	113 (42.8)
Educational status	Unable to write and read	30 (34.1)	28 (15.9)	58 (22.0)
	Primary school	20 (22.7)	48 (27.3)	68 (25.8)
	Secondary school	24 (27.3)	67 (38.1)	91 (34.5)
	Diploma and above	14 (15.9)	33 (18.8)	47 (17.8)
Marital status	Married	75 (85.2)	166 (94.3)	241 (91.3)
	Single	11 (12.5)	8 (4.5)	19 (7.2)
	Divorced	2 (2.3)	2 (1.1)	4 (1.5)
Occupational status	Housewife	47 (53.4)	89 (50.6)	136 (51.5)
	Civil servant	8 (9.1)	30 (17.0)	38 (14.4)
	Private worker	15 (17.0)	29 (16.5)	44 (16.7)
	Farmer	9 (10.2)	18 (10.2)	27 (10.2)
	Student	9 (10.2)	10 (5.7)	19 (7.2)
Sex of newborns	Male	41 (46.6)	94 (53.4)	135 (51.1)
	Female	47 (53.4)	82 (46.6)	129 (48.9)

#### Antepartum factors of study participants

Seventy-six (86.4%) cases and 159 (90.3%) controls their mothers were had antenatal care follow up and 11 (12.5%) cases and 6 (3.4%) controls their mothers were had preeclampsia. Two (2.3%) cases and 5 (2.8%) controls their mothers were with the complication of polyhydramnios and 6 (6.8%) cases and 3 (1.7) controls their mothers were had oligohydramnios as a complication. Three (3.4%) cases and 5 (2.8%) controls their mothers were anemic patients and 8 (9.1%) cases and 11 (6.3%) controls their mothers were had maternal infection. Three (3.4%) cases their mothers had experience of having history of smoking and 10 (11.4%) cases and 34 (19.3%) controls had history abortion. Forty-three (48.9%) cases and 64 (36.4%) controls were prim parous parity.

#### Distributions of intrapartum and fetal factors among participants

Twenty-three (26.1%) cases and 36 (20.5%) controls their mothers were had experience of prolonged labor and 7 (8.0%) cases and 8 (4.5%) controls their mothers were had prolapsed cord as a complication of labor. Sixty-three (71.6%) cases and 135 (76.7%) controls their mothers were delivered spontaneously and 78 (88.6%) cases and 158 (89.8%) controls their mothers were had vertex presentation. Twenty-one (23.9%) cases and 9 (5.1%) controls their mothers were had meconium stained on pelvic examination. Forty-three (48.9%) cases and 19 (10.5%) controls were low birth weight and 49 (55.7%) cases and 40 (22.7%) and controls were preterm (Table 2).

**Table 2:** Distribution of intrapartum and fetal factors of cases and controls attending public hospitals of Central zone, Tigray Region, North Ethiopia 2018

Variables	Category	Cases	Controls	Total
		n = 88 (%)	n = 176 (%)	
Prolonged labor	Yes	23 (26.1)	36 (20.5)	59 (23.3)
	No	65 (73.9)	140 (79.5)	205 (77.7)
Prolapsed cord	Yes	7 (8.0)	8 (4.5)	15 (5.7)
	No	81 (92.0)	168 (95.5)	249 (94.3)
Place of delivery	Hospital	74 (84.1)	163 (92.6)	237 (89.9)
	Other than study hospitals	14 (15.9)	13 (7.4)	27 (10.2)
Mode of delivery	Spontaneous	63 (71.6)	135 (76.7)	198 (75.0)
	Instrumental	9 (10.2)	14 (8.0)	23 (8.7)
	Cesarean section	16 (18.2)	27 (15.3)	43 (16.3)
Presentation	Vertex	78 (88.6)	158 (89.8)	236 (89.4)
	Breech and others	10 (11.4)	18 (10.2)	28 (10.6)
History sedation	Yes	17 (19.3)	35 (19.9)	52 (19.7)
	No	71 (80.7)	141 (80.1)	212 (80.3)
Muonium stained	Yes	21 (23.9)	9 (5.1)	30 (11.4)
	No	67 (76.1)	167 (94.9)	234 (88.6)
CPD	Yes	4 (4.5)	5 (2.8)	9 (3.4)
	No	84 (95.5)	171 (97.2)	255 (96.6)
PROM	Yes	17 (19.3)	43 (24.4)	60 (22.7)
	No	71 (80.7)	133 (75.6)	204 (77.3)
Acidosis	Yes	1 (1.1)	2 (1.1)	3 (1.1)
	No	87 (98.9)	174 (98.9)	261 (98.9)
Professionals	Doctors	31 (35.2)	55 (31.3)	86 (32.6)
	Midwifes	55 (62.5)	121 (68.8)	176 (66.7)
	Nurses	2 (2.3)	0 (0)	2 (0.8)
Placental abruption	Yes	2 (2.3)	1 (0.6)	3 (1.1)
	No	86 (97.7)	175 (99.4)	261 (98.9)
Weight of newborn (kg)	< 2.5	43 (48.9)	19 (10.5)	62 (23.5)
	≥ 2.5	45 (51.1)	157 (89.2)	202 (76.5)
GA (weeks)	< 37	49 (55.7)	40 (22.7)	89 (33.7)
	≥ 37	39 (44.3)	136 (77.3)	175 (66.3)

#### Risk factors of birth asphyxia

As showed from the result of bivariate analysis, 13 variables did show a significant association with birth asphyxia at 25% level of significance. Multivariable logistic regression was done by taking 13 variables into account simultaneously. The backward elimination method of regression was used to assess the confounding.

Educational status of mothers were showed significant association with birth asphyxia. The odds of unable to read and write were 6 times higher compared to those who were educated above diploma [AOR = 6; 95% CI (1.51, 23.80)].

Parity was significantly associated with birth asphyxia. This study showed that those who were primiparous 3 times higher risk than those who were multiparous to effect birth asphyxia [AOR = 3.10; 95% CI (1.51, 6.38)].

Antepartum hemorrhage was significantly associated with birth asphyxia. Mothers who had antepartum hemorrhage had 12 times higher risk than those who were not had antepartum hemorrhage to the outcome of birth asphyxia [AOR = 12; 95% CI (2.29, 63.11)].

Status of meconium stained had significant association with the outcome variable of birth asphyxia. Those who had meconium stained were 7.9 times higher risk than were not had meconium stained to birth asphyxia [AOR = 7.88; 95% CI (2.92, 21.29)].

Preterm babies were 2.2 times higher risk than term developing birth asphyxia [AOR = 2.20; 95% CI (1.02, 4.76)]. Similarly, the weight of the neonate had also a significant association with birth asphyxia. Low birth weight neonates were 6.9 times higher at risk than normal weight as determinant of birth asphyxia [AOR = 6.9; 95% CI (3.01, 15.81)] (Table 3).

**Table 3:** Bivariate and multivariable logistic regression among factors of cases and controls attending public hospitals of Central zone, Tigray Region, North Ethiopia 2018

Variables	Category	Cases	Controls	COR (95% CI)	AOR (95% CI)
		n = 88 (%)	n = 176 (%)		
Residence	Urban	42 (47.7)	105 (59.7)	0.455 (0.270, 0.766)	1.209 (0.558, 2.024)
	Rural	46 (53.3)	71 (40.3)	1	1
Educational status	Unable to write and read	30 (34.1)	28 (15.9)	2.571 (0.918–7.20)	6 (1.512–23.805)*
	Primary school	20 (22.7)	48 (27.3)	2.496 (1.003–6.207)	2.54 (0.807–7.999)
	Secondary school	24 (27.3)	67 (38.1)	0.982 (0.435–2.217)	0.908 (0.318–2.593)
	Diploma and above	14 (15.9)	33 (18.8)	1	1
Parity	Primiparous	43 (48.9)	64 (36.4)	1.672 (0.995–2.809)	3.103 (1.510–6.377)*
	Multiparous	45 (51.1)	112 (63.6)	1	1
Type of pregnancy	Single	79 (89.8)	170 (96.6)	1	1
	Twin and above	9 (10.2)	6 (3.4)	3.228 (1.111–9.381)	1.534 (0.335–7.036)
Maternal hypertension	Yes	5 (5.7)	3 (1.7)	3.474 (0.811–14.88)	2.952 (0.476–18.313)
	No	83 (94.3)	173 (98.3)	1	1
Antepartum hemorrhage	Yes	8 (9.1)	3 (1.7)	5.767 (1.49–22.314)	12.032 (2.294–63.11)*
	No	80 (90.9)	173 (98.3)	1	1
Preeclampsia	Yes	11 (12.5)	6 (3.4)	4.048 (1.44–11.343)	1.386 (0.304–6.314)
	No	77 (87.5)	170 (90.6)	1	1
Place of delivery	Hospital	74 (84.1)	163 (92.6)	0.422 (0.189–0.941)	0.322 (0.108–0.903)
	Other than study hospitals	14 (15.9)	13 (7.4)	1	1
History of abortion	Yes	10 (11.4)	34 (19.3)	2.369 (1.124–4.992)	0.7133 (0.254–2.003)
	No	78 (88.6)	142 (80.7)	1	1
Oligohydramnios	Yes	6 (6.8)	3 (1.7)	4.220 (1.030–17.29)	2.571 (0.498–13.285)
	No	82 (93.2)	173 (98.3)	1	1
Weight of newborn (kg)	< 2.5	43 (48.9)	19 (10.5)	7.896 (4.191–14.87)	6.9 (3.011–15.812)*
	≥ 2.5	45 (51.1)	157 (89.2)	1	1
GA (weeks)	< 37	49 (55.7)	40 (22.7)	4.272 (2.468–7.395)	2.205 (1.022–4.758)*
	≥ 37	39 (44.3)	136 (77.3)	1	1
Muonium stained	Yes	21 (23.9)	9 (5.1)	5.816 (2.534–13.35)	7.88 (2.917–21.289)*
	No	67 (76.1)	167 (94.9)	1	1

The symbol (*) indicated that these factors had significant association with birth asphyxia

### Discussion

This study was aimed to assess risk factors of birth asphyxia in order to tackle the burden of the disease and its associated problems. It has attempted to look the determinants of birth asphyxia by incorporating as many risk factors as possible.

This study showed that illiterate mothers were significant with birth asphyxia. Unable to read and write were 6 times higher compared to those who were educated above diploma. This result is consistent with a study conducted in Nepal and Indonesia [18, 20] which showed that illiteracy was culprits of birth asphyxia. This may be due to maternal illiteracy is a very broad indicator of poor socio-economic conditions associated with consequent malnutrition, frequent pregnancies and also influence care seeking during antepartum period.

Birth weight was significant association to birth asphyxia. Low birth weight was 6.9 times more likely to be asphyxiated than normal weight (≥ 2500 g). This finding is similar with studies conducted in Pakistan and Thailand [19, 21] presented that low birth weight was a risk factors of birth asphyxia. This may be due to the fact that low birth weight was developed due to maternal complication like hypertension, diabetes mellitus that present pre-conception or antepartum.

Preterm babies were 2.2 times more likely to be asphyxiated than term babies. This study is in line with a study conducted in Jordan discovered that preterm babies had risk of development birth asphyxia [20, 22]. This may be due to premature infants are more susceptible to ischemia due to incomplete blood brain barrier formation. Moreover, it may be due to the fact that preterm babies face multiple morbidities including organ system, immaturity specially lung immaturities causing respiratory failure.

Meconium stained mothers had a significant association with birth asphyxia. Those who had meconium stained were 7.9 times higher risk than were not had meconium stained to birth asphyxia. This study is in line with other previous study [19]. In healthy, well oxygenated fetuses, this diluted meconium is readily cleared from the lungs by normal physiological mechanism, however in few cases meconium aspiration syndrome occurs.

Parity had significant association with birth asphyxia. Those who were prim parous 3 times higher risk than those who were multiparous to effect birth asphyxia. This study is consistent with other studies [23, 24]. This may be due to the fact that prim parous are often ignorant of the demands of pregnancy and often neglect regular attendance to antenatal care. This may result in complications that lead to perinatal asphyxia. However, socioeconomic and cultural factors may also contribute for the same.

Antepartum hemorrhage had significant association with birth asphyxia. Mothers who had antepartum hemorrhage had 12 times higher risk than those who were not had antepartum hemorrhage to the outcome of birth asphyxia. This study is in line with studies reported previously [17]. This could be due to the fact in the antepartum bleeding, there is decreased blood flow from mother to placenta so the hypoxemia can occur in the fetus. This condition can lead to perinatal asphyxia if the transfusion to the mother or delivery is postpone.

### Conclusion

Birth asphyxia is one of the worldwide problem of neonates. It arises different complications, if the cases left untreated and leads to death. There are different variables which culprits of birth asphyxia. In this study, maternal illiteracy, prim parous, low birth weight, preterm delivery and meconium stained amniotic fluid were the risk factors of birth asphyxia. Most of these variables are preventable by holistic care of pregnancy, labor and delivery and post-natal care.

## Limitation

- This study is quantitative; it was better if qualitative approach was also employed to investigate in detail on extra determinant factors of birth asphyxia.
- The study was done in single one region of Ethiopia. It is difficult to generalize for the whole country with small sample.
- This study also subjected to recalling bias of mothers when they remembered their previous history.

## Abbreviations

AOR: adjusted odd ratio
COR: crudes odd ratio
SPSS: Statistics Package for Social Science
WHO: World Health Organization
TRHB: Tigray Regional Health Bureau
